# Pineal Gland Volume in Major Depressive and Bipolar Disorders

**DOI:** 10.3389/fpsyt.2020.00450

**Published:** 2020-05-20

**Authors:** Tsutomu Takahashi, Daiki Sasabayashi, Murat Yücel, Sarah Whittle, Valentina Lorenzetti, Mark Walterfang, Michio Suzuki, Christos Pantelis, Gin S. Malhi, Nicholas B. Allen

**Affiliations:** ^1^Department of Neuropsychiatry, University of Toyama School of Medicine, Toyama, Japan; ^2^School of Psychological Sciences, Turner Institute for Brain and Mental Health, Monash University, Clayton, VIC, Australia; ^3^Melbourne Neuropsychiatry Centre, Department of Psychiatry, The University of Melbourne and Melbourne Health, Melbourne, VIC, Australia; ^4^Faculty of Health Sciences, School of Psychology, Australian Catholic University, Melbourne, VIC, Australia; ^5^Department of Neuropsychiatry, Royal Melbourne Hospital, Melbourne, VIC, Australia; ^6^Florey Institute of Neuroscience and Mental Health, University of Melbourne, Melbourne, VIC, Australia; ^7^Discipline of Psychological Medicine, Northern Clinical School, University of Sydney, Sydney, NSW, Australia; ^8^CADE Clinic, Department of Psychiatry, Royal North Shore Hospital, Sydney, NSW, Australia; ^9^Department of Psychology, University of Oregon, Eugene, OR, United States

**Keywords:** pineal gland, melatonin, magnetic resonance imaging, major depressive disorder, bipolar disorder

## Abstract

Abnormal melatonin secretion has been demonstrated in patients with affective disorders such as major depressive disorder (MDD) and bipolar disorder (BD). However, magnetic resonance imaging (MRI) studies that previously investigated the volume of the pineal gland, which regulates circadian rhythms by secreting melatonin, in these patients reported inconsistent findings. The present study employed MRI to examine pineal gland volumes and pineal cyst prevalence in 56 MDD patients (29 currently depressed and 27 remitted patients), 26 BD patients, and matched controls (33 for MDD and 24 for BD). Pineal volumes and cyst prevalence in the current MDD, remitted MDD, and BD groups did not significantly differ from those of the healthy controls. However, pineal gland volumes were significantly smaller in the current MDD subgroup of non-melancholic depression than in the melancholic MDD subgroup. Interestingly, pineal volumes correlated negatively with the severity of *loss of interest* in the current MDD group. Medication and the number of affective episodes were not associated with pineal volumes in the MDD or BD group. While these results do not suggest that pineal volumes reflect abnormal melatonin secretion in affective disorders, they do point to the possibility that pineal abnormalities are associated with clinical subtypes of MDD and its symptomatology.

## Introduction

Hormonal evidence has suggested abnormal melatonin secretion in patients with affective disorders, such as major depressive disorder (MDD) and bipolar disorder (BD), which may contribute to the circadian rhythm dysfunctions commonly observed in these patients (1, 2). While low melatonin secretion irrespective of the mood status (i.e., manic, depressive, and euthymic) in BD appears to support its role as a trait marker (2, 3), previous findings on serum melatonin levels in MDD have been inconsistent (i.e., decreased, normal, or even increased) (1), which may be partly attributed to the heterogeneity of MDD (e.g., melancholic *vs.* atypical subtypes) (4). Previous studies also suggested different alterations in the timing of melatonin secretion that were dependent on the mood status in MDD and BD patients (1, 2). These findings suggest different roles for melatonin abnormalities in the diagnosis (MDD *vs.* BD), MDD subtypes, and mood status of affective disorders.

To date, only a few magnetic resonance imaging (MRI) studies have examined the pineal gland, a neuroendocrine organ involved in circadian regulation through melatonin secretion (5, 6), in affective disorders. Although not consistently replicated, current evidences generally support the notion that the pineal volume, especially its parenchymal (i.e., non-cystic) volume, likely reflects melatonin levels or melatonin secretion patterns for both healthy subjects (7, 8) and patients with affective disorders (9). A recent study demonstrated smaller pineal volumes and a higher prevalence of pineal cysts, which asymptomatically exist in 20%–40% of healthy adults (10, 11), in MDD patients than in healthy controls (12). However, normal pineal volumes have also been reported in MDD (13) and BD (13, 14) patients, in whom pineal volumes were not related to clinical symptoms (9). These inconsistent findings may be partly attributed to different imaging techniques and exclusion criteria for pineal cysts; previous studies (13, 14) estimated pineal volumes two-dimensionally and also excluded patients with pineal cysts. The heterogeneity of MDD cohorts and/or different mood status (e.g., depressive or euthymic) between studies also appear to have biased the findings obtained; however, this hypothesis warrants further study on a well-defined group of MDD patients with different subtypes and illness stages.

The present MRI study examined pineal volumes and pineal cyst prevalence in patients with MDD (currently depressed and euthymic subgroups) and BD and compared them with those in matched controls. The influence of clinical characteristics (e.g., medication, mood status, and symptom severity, and the melancholic *vs.* non-melancholic subtypes of MDD) on pineal volumes in the patient groups was also investigated. Based on previous hormonal and neuroimaging findings that support the heterogeneity of MDD and potential role of melatonin in the mood status, we predicted that MDD but not BD patients have smaller pineal glands and a higher prevalence of pineal cysts, at least in specific subtypes or mood status, which may be associated with symptom severity.

## Materials and Methods

### Participants

Fifty-six MDD patients, 26 BD patients, and 57 healthy matched subjects were included. The local Internal Review Boards (The Prince of Wales Hospital and University of New South Wales research ethics committees and Mental Health Research & Ethics Committee, Melbourne Health, Melbourne, Australia) approved the study protocol, and all study participants provided written informed consent in accordance with the Declaration of Helsinki. Sample characteristics (**Tables 1** and **2**) and inclusion/exclusion criteria were described previously for the MDD (15–17) and BD (18–20) cohorts, who were screened for head trauma, neurological illness, substance misuse, or other serious physical diseases.

**Table 1:** Demographic/clinical characteristics and brain measurements of the major depression cohort.

	Controls	cMDD	rMDD	Group comparisons^a^
	(*N* = 33)	(*N* = 29)	(*N* = 27)	
Age (years)	34.0 ± 9.9	32.5 ± 8.3	35.1 ± 10.0	*F* (2, 86) = 0.52, *p* = 0.595
Male/female	12/21	7/22	9/18	Chi-squared = 1.13, *p* = 0.568
Current IQ	111.1 ± 10.9	104.9 ± 8.7	111.4 ± 9.9	*F* (2, 85) = 4.03, *p* = 0.021; not significant (Scheffé's test)
Premorbid IQ	111.6 ± 12.3	107.5 ± 11.4	111.7 ± 8.9	*F* (2, 86) = 1.41, *p* = 0.250
Age of onset	–	21.1 ± 8	26.0 ± 9.4	*F* (1, 54) = 4.56, *p* = 0.037; cMDD < rMDD
Number of episodes	–	3.7 ± 3.4	3.1 ± 2.6	*F* (1, 38) = 0.37, *p* = 0.547
First episode/recurrent	–	7/22	–	*-*
Melancholic/non-melancholic^b^	–	10/18	–	*-*
Medication past 6 months: yes/no	–	21/6	12/13	Chi-squared = 4.96, *p* = 0.026
Current anxiety disorder: yes/no	–	18/10	4/23	Chi-squared = 14,02 *p <* 0.001
Beck Depression Inventory	3.6 ± 4.1	36.8 ± 8.9	13.0 ± 11.7	*F* (2, 86) = 120.57, *p <* 0.001; cMDD > rMDD > controls
MASQ general distress	27.9 ± 8.3	50.5 ± 7.8	40.4 ± 10.3	*F* (2, 81) = 49.21, *p <* 0.001; cMDD > rMDD > controls
MASQ general depression	19.5 ± 7.2	47.3 ± 9.2	35.0 ± 11.7	*F* (2, 82) = 66.85, *p <* 0.001; cMDD > rMDD > controls
MASQ general anxiety	16.4 ± 6.4	32.3 ± 8.7	24.7 ± 7.7	*F* (2, 82) = 32.31, *p <* 0.001; cMDD > rMDD > controls
MASQ anxious arousal	22.0 ± 4.4	42.0 ± 12.2	28.9 ± 7.7	*F* (2, 79) = 40.47, *p <* 0.001; cMDD > rMDD > controls
MASQ high positive affect	81.1 ± 14.3	43.6 ± 13.5	65.0 ± 12.4	*F* (2, 80) = 57.19, *p <* 0.001; cMDD < rMDD < controls
MASQ loss of interest	14.7 ± 5.0	31.6 ± 6.4	23.5 ± 6.8	*F* (2, 82) = 58.68, *p <* 0.001; cMDD > rMDD > controls
PANAS positive affect	32.9 ± 7.3	21.6 ± 6.5	28.7 ± 8.0	*F* (2, 82) = 18.57, *p <* 0.001; cMDD < rMDD, controls
PANAS negative affect	11.2 ± 1.6	21.2 ± 8.5	14.2 ± 4.7	*F* (2, 83) = 24.98, *p <* 0.001; cMDD > rMDD, controls
Total pineal volume [mm^3^ (Cohen's *d* relative to controls)]	145.2 ± 84.9	119.2 ± 51.5 (-0.37)	119.7 ± 53.7 (-0.36)	*F* (2, 81) = 0.65, *p* = 0.526
Pineal parenchymal volume [mm^3^ (Cohen's *d* relative to controls)]	138.8 ± 71.7	115.0 ± 45.1 (-0.40)	116.5 ± 48.2 (-0.37)	*F* (2, 81) = 0.84, *p* = 0.435
Cyst (≥ 2 mm) [*N*(%)]	9 (27.3%)	11 (37.9%)	7 (25.9%)	Chi-squared = 1.19, *p* = 0.553
Small cystic change (< 2 mm) [*N*(%)]	7 (21.2%)	5 (17.2%)	7 (25.9%)	Chi-squared = 0.63, *p* = 0.730
Intracranial volume (cm^3^)	1493 ± 143	1477 ± 138	1470 ± 150	*F* (2, 85) = 0.20, *p* = 0.816^c^

Values represent means ± SD.

cMDD, currently depressed patients; MASQ, Mood and Anxiety Symptom Questionnaire; PANAS, Positive and Negative Affect Schedule; rMDD, remitted depressed patients.

^a^Differences between the degree of freedom across measures were due to missing data.

^b^Assessed only for cMDD patients. Information missing for one patient.

^c^ANCOVA with age as a covariate and group as a between-subject factor.

**Table 2:** Demographic/clinical characteristics and brain measurements of the bipolar disorder cohort.

	Controls	Bipolar disorder	Group comparisons
	(*N* = 24)	(*N* = 26)	
Age (years)	38.7 ± 11.1	38.4 ± 10.9	*F* (1, 48) = 0.01, *p* = 0.928
Male/female	7/17	8/18	Chi-squared = 0.02, *p* = 0.902
NART-estimated IQ^a^	115.1 ± 9.6	113.8 ± 7.1	*F* (1, 47) = 0.28, *p* = 0.597
Education (years)	14.6 ± 2.1	14.7 ± 2.8	*F* (1, 48) = 0.02, *p* = 0.899
Illness duration (years)	–	13.5 ± 10.1	–
Number of manic episodes	–	8.8 ± 10.2	–
Number of depressive episodes	–	11.1 ± 10.8	–
Lithium dosage (mg, *N* = 12)	–	975 ± 213	–
Valproate dosage (mg, *N* = 12)	–	1437 ± 594	–
Total pineal volume [mm^3^ (Cohen's *d* relative to controls)]	129.8 ± 62.0	121 ± 79.0 (-0.13)	*F* (1, 44) = 0.64, *p* = 0.430
Pineal parenchymal volume [mm^3^ (Cohen's *d* relative to controls)]	126.4 ± 57.6	119.6 ± 76.8 (-0.10)	*F* (1, 44) = 0.60, *p* = 0.442
Cyst (≥ 2 mm) [*N*(%)]	6 (25%)	5 (19.2%)	Chi-squared = 0.24, *p* = 0.623
Small cystic change (< 2 mm) [*N*(%)]	6 (25%)	11 (42.3%)	Chi-squared = 1.67, *p* = 0.197
Intracranial volume (cm^3^)	1461 ± 148	1476 ± 126	*F* (1, 47) = 0.13, *p* = 0.715^b^

Values represent means ± SD.

NART, National Adult Reading Test.

^a^Data missing for one bipolar patient.

^b^ANCOVA with age as a covariate and group as a between-subject factor.

Briefly, the MDD cohort was recruited through an advertisement in the local media and *via* outpatient psychiatric clinics in Melbourne, Australia, and comprised 29 patients currently under a depressive state (cMDD), 27 with a history of MDD but currently in remission (rMDD), and 33 healthy controls with no personal history of neuropsychiatric diseases. All participants underwent clinical and neuropsychological assessments by experienced research psychologists at ORYGEN Youth Health, Melbourne, with the Structured Clinical Interview for DSM-IV (SCID-IV-TR) (21), the Beck Depression Inventory (BDI) (22), Mood and Anxiety Symptom Questionnaire (MASQ) (23), and Positive Affect and Negative Affect Scale (PANAS) (24). The medication status, case history, and comorbid anxiety disorder were also examined. Depression subtype (melancholic *vs.* non-melancholic) was assessed only for the cMDD group; the melancholic depressed patients fulfilled the SCID criteria (21) based on the eight symptoms of the melancholic specifier (i.e., a loss of pleasure, lack of reactivity to usually pleasurable stimuli, distinct quality of depressed mood, mood regularly worse in the morning, insomnia, psychomotor retardation or agitation, significant anorexia or weight loss, and excessive or inappropriate guilt).

Twenty-six patients with bipolar I disorder under euthymic conditions were recruited from the Mood Disorders Unit at the Prince of Wales Hospital, Sydney, Australia, at which research psychiatrists made diagnoses using the SCID-IV patient version (25) supplemented by chart reviews. Twenty-four healthy subjects, screened using the SCID-IV non-patient version (25), were recruited through the advertisement. Ten BD patients had a family history of affective disorders, such as BD (*N* = 3), MDD (*N* = 5), and both (*N* = 2), whereas 12 did not and four had an unknown family illness history. Sixteen BD patients had experienced psychotic symptoms (hallucinations and/or delusions) during affective episodes.

### MRI Acquisition and Data Processing

MR scans of MDD cohort were acquired using a 1.5T Siemens scanner (Magnetom Avanto) at Saint Vincent's Hospital Melbourne, Victoria (16, 17). Structural T1-weighted axial images were obtained using the following parameters: time to echo = 2.3 ms, time repetition = 2.1 ms, flip angle = 15°, matrix size = 256 × 256, voxel dimension = 1 × 1 × 1 mm.

T1-weighted images of BD cohort were acquired in the coronal orientation using a 1.5-T GE Signa scanner located at the Royal Prince Alfred Hospital, Sydney, Australia, with a fast-spoiled gradient echo sequence (time to echo = 5.3 ms, time repetition = 12.2 ms, flip angle = 25°, matrix size = 256 × 256, and voxel dimensions = 0.98 × 0.98 × 1.6 mm) (18, 19).

Brain images were realigned in three dimensions using Dr. View software (Infocom, Tokyo, Japan), and then reconstructed into 1.0-mm (MDD cohort)- or 0.98-mm (BD cohort)-thick entire contiguous coronal images. Voxels were segmented into brain tissue components and cerebrospinal fluid (CSF) based on the signal-intensity histogram distribution of each T1-weighted image (26, 27). The intracranial volume (ICV) was measured on a sagittal reformat of the original 3D data (28), and did not significantly differ among the groups examined (**Tables 1** **and** **2**).

### Pineal Gland Measurements

**Figure 1:**
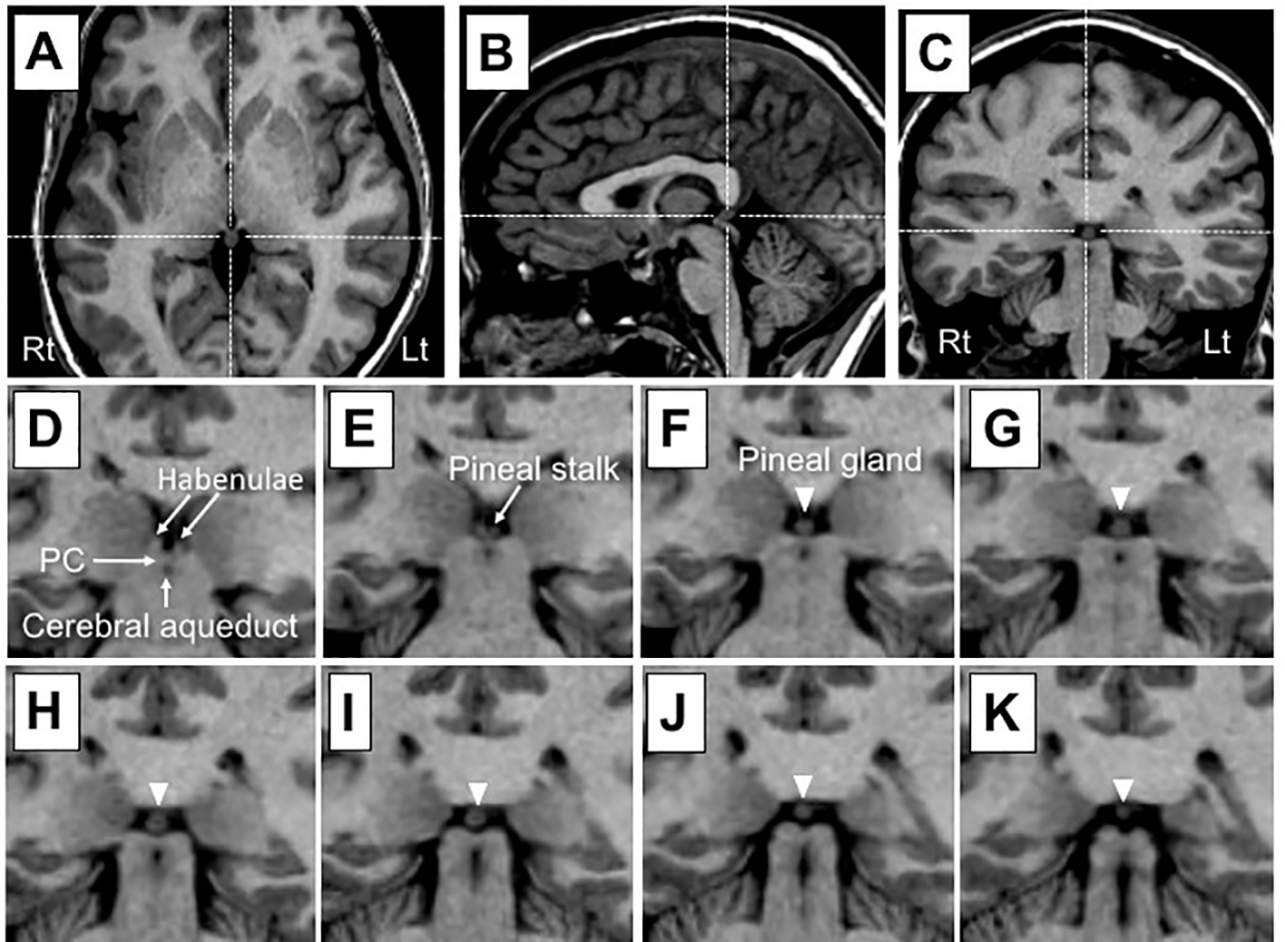
Sample T1 images of the pineal gland in a subject with a small cystic change. Dotted lines in **(A)** (axial), **(B)** (sagittal), and **(C)** (coronal) show pineal gland coordinates. The pineal gland (arrowhead) and neighboring anatomical landmarks are shown on consecutive 1-mm-thick coronal slices from an anterior **(D)** to posterior **(K)** direction. The pineal gland is located posterior to the habenular nucleus and may be readily delineated on voxels as a brain tissue component largely surrounded by cerebrospinal fluid, except at its attachment to the stalk. The pineal stalk was excluded from the measurement of pineal gland volumes. PC, posterior commissure.

As reported previously (26, 27), one rater (TT), who was blinded to subject identities, manually traced the pineal gland, a small pinecone-shaped endocrine gland surrounded by CSF, except at the connection to the habenulae (**Figure 1**), on consecutive coronal slices. The parenchyma (i.e., segmented brain tissue component) of the pineal gland and internal cystic changes (pineal cyst ≥ 2 mm or small cystic change < 2 mm in diameter), observed as circular areas of iso-intensity relative to CSF (10), were differentiated by the signal intensity of each image. Therefore, we obtained total (cyst included) and parenchymal (non-cystic) pineal volumes. Intra- (TT) and inter-rater (TT and DS) intraclass correlation coefficients in a subset of 12 randomly selected brains (7 from the MD cohort and 5 from the BD cohort) were all > 0.90.

### Statistical Analysis

Demographic and clinical differences between groups (cMDD *vs.* rMDD *vs.* controls, BD *vs.* controls) were assessed using a one-way analysis of variance (ANOVA) or the chi-squared test.

An analysis of covariance (ANCOVA) was performed on pineal volumes (total and parenchymal volumes) using age and ICV as covariates and group (cMDD *vs.* rMDD *vs.* controls, BD *vs.* controls) and gender as between-subject factors. The same ANCOVA model was used for assessing the effect of MDD subgroups [i.e., melancholic features (information available only for cMDD patients), co-morbid anxiety disorder, medication status, and first-episode or recurrent cMDD group] on the pineal volumes. Scheffé's test was used to follow-up any significant main effects or interactions. Group differences in the prevalence of pineal cysts (≥ 2 mm) and small cystic changes (< 2 mm) were examined using the chi-squared test. Relationships between pineal volumes and clinical variables were investigated by Pearson's partial correlation coefficients, with adjustments for age and ICV. To reduce the rate of Type I errors due to multiple comparisons, only parenchymal (non-cystic) volumes, which more accurately reflect the levels of melatonin secreted than total pineal volumes (7, 8), were used in correlational analyses. Pineal volumes and clinical variables (number of episodes, medication, and symptom measures) were log-transformed for statistical analyses because of their skewed distribution (tested by the Kolmogorov– Smirnov test). A *p*-value of < 0.05 was considered to be significant.

## Results

### Demographic and Clinical Characteristics

The MDD (**Table 1**) and BD (**Table 2**) groups were matched for age, gender, and intelligence or education with healthy controls. The cMDD group was characterized by an earlier onset age, higher proportion of medicated patients, higher rate of comorbid anxiety disorder, and more severe depressive/anxiety symptoms than the rMDD group (**Table 1**).

### Pineal Gland Volume

**Figure 2:**
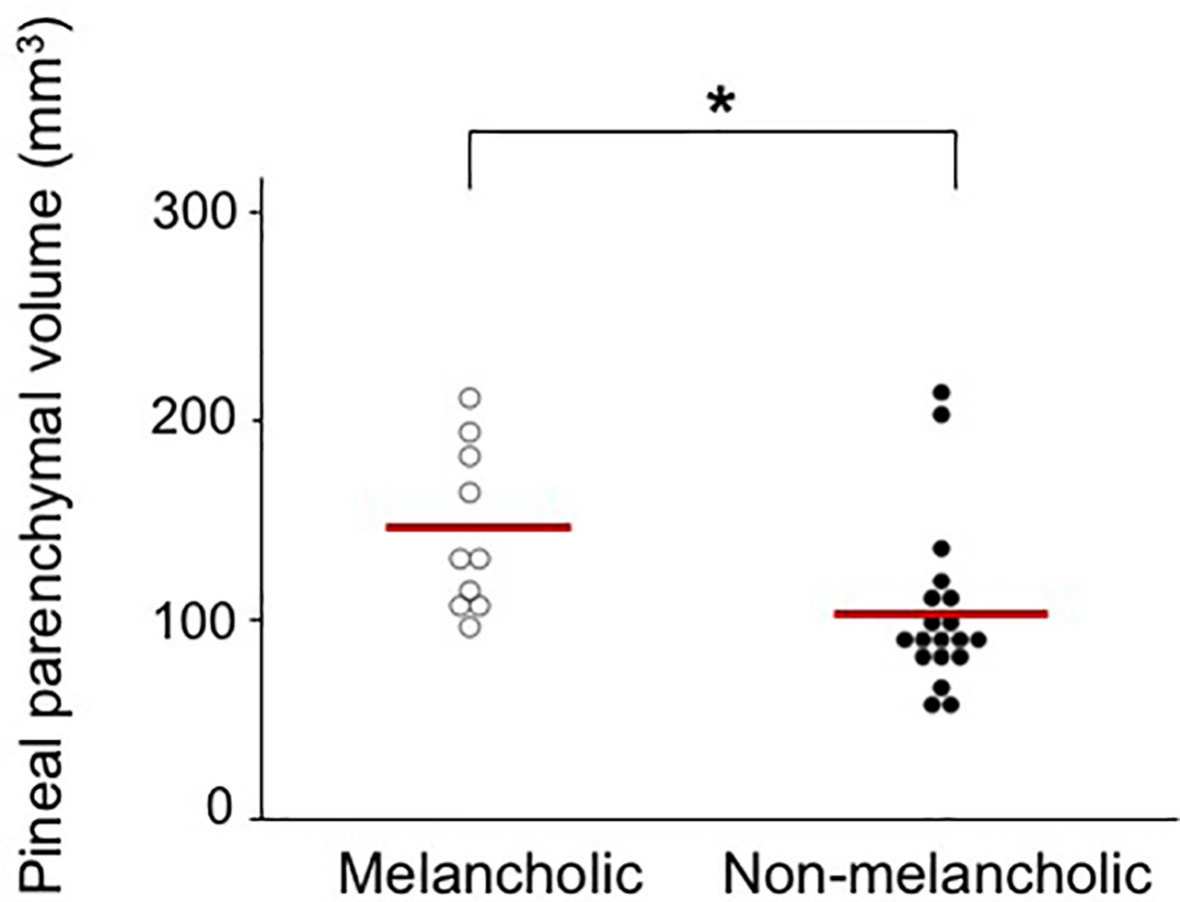
Absolute pituitary parenchymal volume in currently depressed patients with melancholic (142.0 ± 40.7mm^3^, Cohen's *d* relative to controls = 0.05) and non-melancholic (101.9 ± 42.4 mm^3^, Cohen's *d* relative to controls = -0.63) subtypes. Scheffé's test: *p < 0.05.

Total and parenchymal pineal volumes in the cMDD and rMDD groups did not differ significantly from those of the healthy controls without any significant effect involving gender. However, these MDD groups exhibited non-significant pineal atrophy to the same degree as compared with healthy controls (Cohen's *d* relative to controls = -0.36 to -0.40) (**Table 1**). Comparisons between cMDD patients with and without melancholic features showed a significant difference in total [*F* (1, 24) = 4.98, *p* = 0.035] and parenchymal [*F* (1, 24) = 4.74, *p* = 0.040] volumes; the non-melancholic group had a significantly smaller volume than the melancholic group (*p* = 0.014 for both total and parenchymal volumes) (**Figure 2**). Total and parenchymal pineal volumes did not significantly differ between MDD patients with and without co-morbid anxiety disorder, those with and without medication in the past 6 months, or first-episode and recurrent cMDD patients.

No significant differences were observed in total (Cohen's *d* = -0.13) or parenchymal (Cohen's *d* = -0.10) pineal volumes between the BD group and healthy controls (**Table 2**). No significant effect involving gender was found. Furthermore, total and parenchymal pineal volumes did not significantly differ between the patient subgroups based on psychotic symptoms, family history, and medication status (lithium and valproate).

### Pineal Cyst and Small Cystic Change

No significant differences were observed in the prevalence of pineal cysts and small cystic changes between the groups in the MDD (**Table 1**) and BD (**Table 2**) cohorts.

### Correlational Analyses

**Figure 3:**
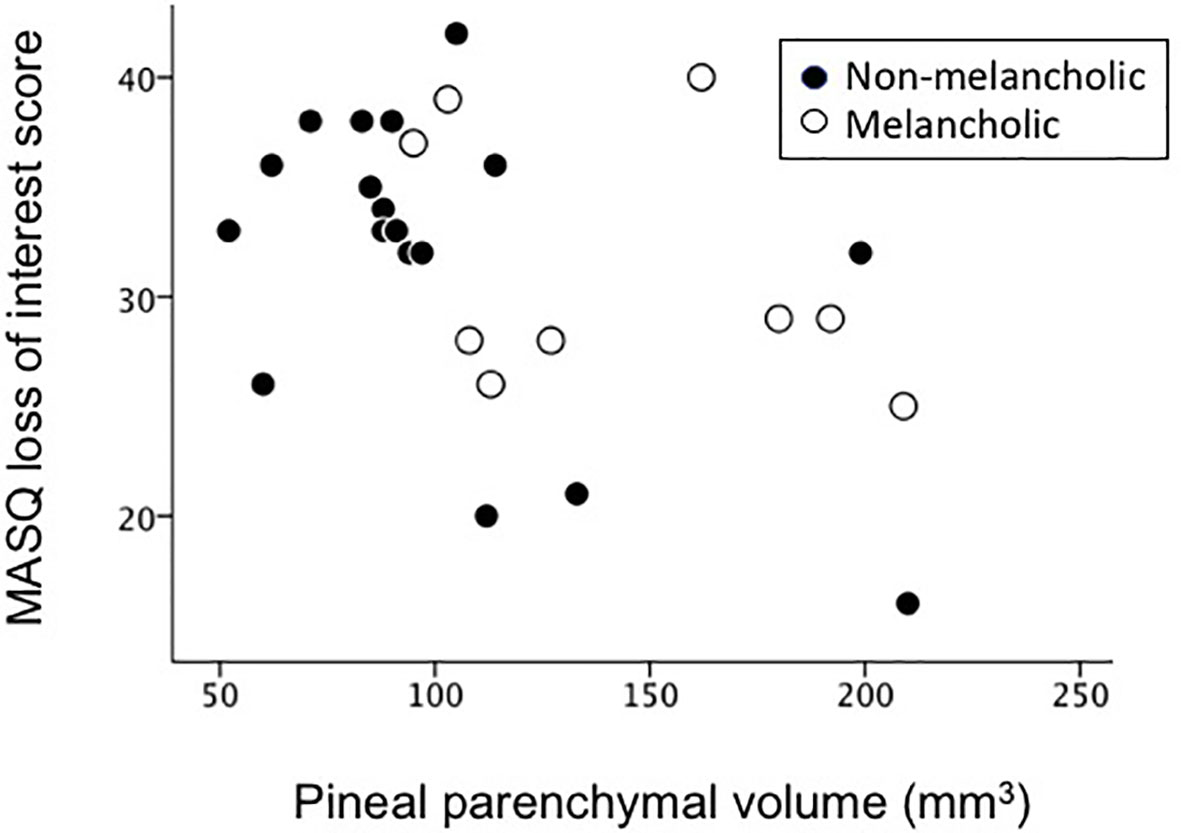
Relationship between pineal parenchymal volumes and Mood and Anxiety Symptom Questionnaire (MASQ) loss of interest scores in currently depressed patients.

Pineal parenchymal volumes in cMDD patients negatively correlated with the MASQ loss of interest score (*r* = -0.571, *p* = 0.002; **Figure 3**) even after the Bonferroni correction for multiple comparisons [10 clinical variables in two groups; *p* < 0.0025 (0.05/20)], but not with the number of episodes, total BDI score, and other MASQ and PANAS subscale scores. These clinical variables did not correlate with pineal parenchymal volumes in the rMDD group.

In the BD cohort, pineal parenchymal volumes did not correlate with illness duration, the number of manic/depressive episodes, or medication dose (lithium and valproate).

## Discussion

In the present study, no significant differences were observed in pineal volumes or cyst prevalence between the currently depressed and remitted MDD subgroups, bipolar I disorder group, and their matched controls. However, pineal volumes were specifically reduced in non-melancholic MDD patients and also negatively correlated with the severity of *loss of interest* in MDD patients under an active depressive state, thereby supporting the role of pineal abnormalities in certain clinical aspects of MDD.

Consistent with previous findings reported by Fındıklı et al. (13), the pineal volumes of MDD patients in the present study did not significantly differ from those of control subjects. However, these findings and the present results suggest non-significant pineal atrophy in MDD with the degree of a small to medium effect size (Cohen's *d* relative to controls = approximately -0.4 for both studies; see **Table 1**), which was smaller than, but similar to a recent study by Zhao et al. (9) (Cohen's *d* = -0.57) that reported significantly decreased pineal volumes in MDD. While Fındıklı et al. (13) estimated pineal volumes using a two-dimensional approximation formula (29) in a relatively small sample of MDD patients who had no pineal cysts (*N* = 16), Zhao et al. (12) and the present study manually measured true parenchymal (non-cystic) volumes, which appear to reflect pineal function more accurately than total (cyst included) pineal volumes (7, 8), in larger MDD cohorts regardless of the presence or absence of pineal cysts (*N* ≥ 50). Thus, inconsistent pineal findings among studies (i.e., degree of volume reductions in MDD) cannot only be explained by differences in imaging techniques, approaches to pineal cysts, or sample sizes. However, the heterogeneity of MDD discussed below may be relevant.

The present results showing different pineal volumes between the melancholic and non-melancholic subtypes of depression support MDD being a heterogeneous disorder with different phenotypes caused by various neuropathological alterations (30, 31). Since the melancholic features of depression, such as diurnal variations in mood and insomnia (21), imply melatonin abnormalities and related circadian rhythm dysfunctions, the present results showing greater pineal atrophy in the non-melancholic MDD subtype were unexpected. On the other hand, we demonstrated that the severity of *loss of interest*, one of the core factors of melancholic depression (32), correlated with the degree of pineal reduction. While some neuroimaging [e.g., hippocampal atrophy (33) and hypofrontality (34)] and neuroendocrine [e.g., abnormal dexamethasone suppression pattern (35)] findings appear to be associated with the pathophysiology of melancholia, there have been no definitive biological markers of different subtypes of depression (35). However, the present results support pineal abnormalities potentially contributing to the clinical subtype and symptomatology of MDD.

In the present study, the illness stages (i.e., number of episodes, first-episode *vs.* multiple episodes) and mood status (currently depressed or remitted) of MDD patients did not affect pineal volumes, supporting its role as a stable trait marker. This appears to be consistent with previous clinical/hormonal findings showing that altered patterns of melatonin secretion (36) and circadian deregulation (37) were also present in the remission phases of depression, which may be relevant to residual symptoms or vulnerability to relapse. While this structural MRI study did not investigate the mechanisms underlying potential pineal volume changes, pineal dysfunctions in MDD are considered to be primarily caused by serotoninergic and norepinephrinergic deficits (36). However, the pineal pathology of depression has not yet been elucidated in detail; active structural/functional alterations in the pineal gland may occur around the first manifestation of depressive symptoms, while the genetic control of circadian rhythms in mood disorders (37) and animal findings of the significant contribution of intrauterine (maternal) melatonin deprivation to depressive symptoms in adult offspring (38) appear to support its neurodevelopmental aspects. Longitudinal studies on pineal volumes and melatonin secretion are required to clarify the nature of pineal abnormalities in MDD, particularly in the early course of the illness.

In contrast to a previous MRI study showing a higher prevalence of pineal cysts in MDD patients (62%) than in healthy subjects (40%) (12), our cohort had a prevalence of approximately 50% [combined rate of macroscopic cysts (≥ 2 mm in diameter) and small cystic changes (< 2 mm in diameter)] in the MDD and control groups (**Table 1**). Since macroscopic pineal cysts may affect melatonin (39) as well as cortisol (40) secretion profiles, the higher prevalence of pineal cysts may have induced neuroendocrine disturbances and consequent depressive symptomatology (41). However, Zhao et al. (12) did not differentiate pineal cysts and small cystic changes and we were unable to reliably evaluate exact cyst sizes in all cases using MR images due to the partial volume effect; therefore, future studies using higher-resolution images are needed to clarify the role of pineal cysts in the pathophysiology of major depression.

BD patients in the present study showed no abnormal morphological changes (i.e., total or parenchymal volumes and cyst prevalence) in the pineal gland, and the pineal morphology was not associated with clinical variables. Previous MRI studies reported a normal pineal volume in BD (13, 14), suggesting no significant role for pineal volume in the pathophysiology of BD. However, other hormonal studies have suggested that abnormal melatonin secretion is a heritable trait marker of BD (2, 42). Potential treatment effects of melatonin for mood symptoms and relapse prevention in BD (2) also support melatonin dysregulation in these patients. Thus, the normal relationship between the volume of the pineal gland and its secretion of melatonin (7, 8) may be disrupted under pathological conditions, such as BD.

The present study had several limitations. First, we did not assess melatonin levels or circadian rhythms in study participants. Therefore, it currently remains unclear whether the pineal results obtained reflect its function and disturbances in the circadian rhythms of MDD patients. Further, given the association between the pineal activation and physical/mental relaxation (43), potential role of its abnormality in affective disorders needs to be tested in future functional neuroimaging studies. Second, the sample size of the study participants was rather small, especially for each MDD subgroup [e.g., melancholic cMDD group (*N* = 10), first-episode cMDD group (*N* = 7)]. In addition, the information of melancholic/non-melancholic subtype was not available for the rMDD group in this study. It should be also noted that the MDD and BD cohorts were scanned using different scanners/parameters in the present study, which disabled direct comparisons of pineal volumes between the MDD and BD groups. Control matched groups for the MDD and BD groups showed markedly different pineal volumes (**Tables 1** and **2**), which may reflect the influence of the different imaging settings. Thus, our preliminary results need to be replicated in a larger sample of various affective disorders scanned using the same setting. Third, as discussed previously (26), T1-weighted MR images cannot reliably assess pineal calcification, which may be associated with melatonin secretion (44) and treatment responses in bipolar patients (45). Another technical issue is that the error caused by manual measurement cannot be avoided especially for a small structure such as the pineal gland. While the measurement in this study required only minimal manual editing (**Figure 1**) and the inter- and intra-rater reliabilities were rather high (> 0.90), future studies using non-manual automated measurement methods would increase the accuracy of the pineal gland assessment. Finally, while the present study found no effect of medication on pineal volumes in the MDD and BD cohorts, their complete medication data (e.g., lifetime medication) were not available. Since mood stabilizers (46, 47) and antidepressants (48) have been shown to affect melatonin secretion patterns or brain melatonin receptor expression, the potential effects of prolonged medication on pineal morphology/functions warrant further study.

In summary, the present study found that pineal gland volumes were not significantly altered in patients with MDD and BD. These results do not support the association of pineal volumes with abnormal melatonin secretion in affective disorders (1, 2). However, significant reductions were observed in pineal volumes in specific depression subtypes, and these changes correlated with specific symptoms of depression, suggesting an association between pineal abnormalities and clinical subtype and/or symptomatology of major depression.

## Data Availability Statement

The datasets generated for this study will not be made publicly available because we do not have permission to share the data. Requests to access the datasets should be directed to the corresponding author.

## Ethics Statement

The studies involving human participants were reviewed and approved by The Prince of Wales Hospital and University of New South Wales research ethics committees and Mental Health Research & amp; Ethics Committee, Melbourne Health, Melbourne, Australia. The patients/participants provided their written informed consent to participate in this study.

## Author Contributions

MY, MS, CP, GM, and NA conceived the concept for and methodology of the study. TT conducted statistical analyses and wrote the manuscript. MY, SW, VL, MW, GM, and NA recruited subjects and were involved in clinical and diagnostic assessments. TT and DS analyzed MRI data. MY, MS, CP, GM, and NA contributed to the writing and editing of the manuscript. All authors contributed to and have approved the final manuscript.

## Funding

This work was supported in part by JSPS KAKENHI Grant Number No. JP18K07550 to TT, JP18K15509 to DS, and by Health and Labour Sciences Research Grants for Comprehensive Research on Persons with Disabilities from the Japan Agency for Medical Research and Development (AMED) Grant Number 16dk0307029h0003 to MS.

## Conflict of Interest

The authors declare that this research was conducted in the absence of any commercial or financial relationships that may be construed as a potential conflict of interest.
